# Impact of switching from triple therapy to dual bronchodilation in COPD: the DACCORD ‘real world’ study

**DOI:** 10.1186/s12931-022-02037-2

**Published:** 2022-05-02

**Authors:** Claus F. Vogelmeier, Heinrich Worth, Roland Buhl, Carl-Peter Criée, Eva Gückel, Peter Kardos

**Affiliations:** 1 Department of Medicine, Pulmonary and Critical Care Medicine, University Medical Centre Giessen and Marburg, Philipps-University Marburg, Member of the German Centre for Lung Research (DZL), 35043 Marburg, Germany; 2 Facharztforum Fürth, 90762 Fürth, Germany; 3 Pulmonary Department, Mainz University Hospital, 55131 Mainz, Germany; 4 Department of Sleep and Respiratory Medicine, Evangelical Hospital Goettingen-Weende, 37120 Bovenden, Germany; 5 Clinical Research, Respiratory, Novartis Pharma GmbH, 90429 Nürnberg, Germany; 6 Group Practice and Centre for Allergy, Respiratory and Sleep Medicine, Red Cross Maingau Hospital, 60316 Frankfurt am Main, Germany

## Abstract

**Introduction:**

Chronic obstructive pulmonary disease (COPD) guidelines recommend reserving triple therapy of inhaled corticosteroid (ICS), long-acting β_2_-agonist (LABA) and long-acting muscarinic antagonist (LAMA) for patients with exacerbations despite dual therapy. However, many patients receive triple therapy without a clear indication. For these patients, it would be useful to know whether ICS can be withdrawn.

**Methods:**

DACCORD was a longitudinal, non-interventional ‘real-world’ study in three cohorts. This manuscript describes the results of Cohort 3, which recruited patients with COPD who had received triple therapy for ≥ 6 months. Prior to entry, each patient’s physician decided to continue triple therapy, or switch to a LABA/LAMA; patients were then followed for 12 months, with exacerbations and COPD Assessment Test (CAT) data recorded every 3 months. The primary endpoint was the time until COPD worsening, defined as the occurrence of a moderate/severe exacerbation or clinically relevant CAT worsening.

**Results:**

Of the 1192 patients recruited into the study, 967 completed the end-of-study visit and ≥ 2 of the three interim visits, 292 and 675 receiving LABA/LAMA and triple therapy, respectively. Most baseline demographics were similar between the two groups. A lower proportion of patients in the LABA/LAMA group had COPD worsening than with triple therapy (32.5% vs 55.7% at 12 months), with the time to worsening extended in the LABA/LAMA group (hazard ratio 2.004, p < 0.001). In addition, a significantly lower proportion of patients in the LABA/LAMA group exacerbated (18.5% vs 28.7%; p < 0.001), accompanied by a greater improvement from baseline in CAT total score. Overall, fewer patients in the LABA/LAMA group reported adverse events than in the triple therapy group (12.9% vs 15.1%).

**Conclusions:**

These results suggest that in a real world setting physicians are able to identify patients who can be ‘stepped down’ from triple therapy to LABA/LAMA. Following step down, there was no overall decline in COPD—indeed, some patients had better outcomes.

## Introduction

In the management of chronic obstructive pulmonary disease (COPD), the preferred treatment option is the use of bronchodilators—either mono-bronchodilation for milder disease, or a long-acting β_2_-agonist plus a long-acting muscarinic antagonist (LABA/LAMA) in patients with more severe disease [1], with some data supporting the use of LABA/LAMA even as first-line therapy [2]. Triple therapy comprising inhaled corticosteroid (ICS), LABA and LAMA should ideally be reserved for patients with exacerbations despite dual therapy [1], since, although ICS use can bring benefits, this increases the risk (and associated cost) of adverse events such as oral thrush, hoarseness and pneumonia [3]. This is especially relevant since a large proportion of patients receive triple therapy without a clear indication [4–6]. For these patients, it would be useful to know whether ICS can be withdrawn without impacting disease stability.

The 12-month WISDOM randomised controlled trial (RCT) recruited patients with COPD who had severe or very severe airflow limitation and a history of at least one exacerbation, 39% of whom were receiving triple therapy on entry [7]. After a run-in period during which all patients received triple therapy, patients were randomised to either continue triple therapy or to have the ICS gradually withdrawn. Although there was a somewhat greater fall in lung function in the ICS withdrawal arm, there was no difference between groups in COPD exacerbation rate. The 6-month SUNSET RCT recruited patients with COPD who had moderate or severe airflow limitation, a history of no more than one exacerbation in the previous year (66% had not exacerbated), and who had been receiving triple therapy for at least 6 months prior to entry [8]. After a run-in period during which all patients received triple therapy, patients were randomised to either switch to LABA/LAMA or continue triple therapy. As with WISDOM, there was a slightly greater fall in lung function in the ICS withdrawal arm, but no difference between groups in COPD exacerbation rate; importantly, there was an improvement from baseline in health-related quality of life in both arms. These two studies provided an initial indication that step-down from triple therapy to LABA/LAMA could be achieved in patients with COPD. However, as both were RCTs with selected populations, the relevance to clinical practice is limited. A retrospective database study subsequently evaluated this step-down, confirming that discontinuation of ICS from triple therapy was not associated with an overall increased exacerbation risk [9].

DACCORD is a longitudinal, non-interventional real world study that has recruited patients with COPD in three cohorts. The first cohort recruited patients who started or switched to a treatment regimen including the LAMA glycopyrronium prior to entry, with a non-glycopyrronium group as comparator [10–14]; the second cohort recruited patients who either started or switched to a regimen containing a LABA/LAMA fixed-dose combination (FDC), with a non-LABA/LAMA FDC group as comparator [15]. In *post-hoc* analyses of subgroup data from these two cohorts, we evaluated the impact of ICS withdrawal [12], and demonstrated that a step-down from triple therapy to indacaterol/glycopyrronium could be achieved in daily practice without triggering exacerbations or a worsening in health-related quality of life [15]. The third cohort of DACCORD aimed to confirm these previous findings, by specifically recruiting patients who had been receiving triple therapy for ≥ 6 months. The decision was then left to the treating physician as to whether patients were to continue triple therapy or be switched to a LABA/LAMA FDC prior to entry, and these patients were followed up for 12 months.

## Methods

### Trial design

Patients in Cohort 3 of DACCORD were recruited at 85 primary and secondary care practices distributed throughout Germany. For this cohort, eligible patients were adults aged ≥ 40 years who had a confirmed diagnosis of COPD, were included in the Disease Management Program (DMP) for COPD or fulfilled the criteria for inclusion, had been receiving LABA + LAMA + ICS therapy for ≥ 6 months prior to baseline, and had available data on blood eosinophil count (determined ≤ 6 months prior to inclusion). All patients provided written informed consent prior to inclusion. Exclusion criteria were limited to inclusion in the asthma DMP, concomitant asthma or a prior asthma diagnosis, foreseeable problems in follow-up across the study duration, or current RCT participation.

Prior to study entry, each patient’s physician decided to either continue maintenance treatment with triple therapy, or switch the patient to a LABA/LAMA FDC. Given the non-interventional, ‘real-life’ nature of the study, this decision was to have been made by the treating physician based only on the individual circumstances of the patient, and was neither influenced by the fact that patients were included in DACCORD nor by the protocol itself. Specific visits were not mandated by the protocol, but, consistent with usual care in Germany, it was anticipated that data would be recorded approximately every 3 months. At the baseline visit, data collected in internet-based electronic case report forms included: demographics and disease characteristics; differential blood count; prescribed COPD medication; COPD Assessment Test (CAT); exacerbations in the 12 months prior to entry (defined based on prescription of oral steroids and/or antibiotics or hospitalisation); lung function; and the physician’s or patient’s reasons for either switching to a LABA/LAMA FDC or remaining on triple therapy. Data on prescribed COPD medication, exacerbations, and CAT were collected every 3 months, with demographics, disease characteristics, and lung function also recorded at the end of study visit.

The study was registered in the European Network of Centers for Pharmacoepidemiology and Pharmacovigilance (EUPAS4207;) and was approved by the ethics committee of the University of Erlangen-Nürnberg, Germany.

### Outcomes

The primary objective of DACCORD Cohort 3 was to assess the time to worsening of COPD, defined as the occurrence of a moderate/severe exacerbation or of a clinically relevant worsening in health-related quality of life (i.e., an increase from baseline in CAT score of ≥ 2 points) in patients treated with a LAMA/LABA FDC after switching from inhaled triple therapy, versus patients continuing on inhaled triple therapy, assessed over the 1 year follow-up period.

Secondary endpoints included: Time to first occurrence of a moderate or severe exacerbation, and to a clinically relevant worsening of health-related quality of life (worsening from baseline in CAT score of ≥ 2 points); the number and annualised rate of moderate/severe exacerbations; CAT change from baseline, and the percentage of patients with a clinically relevant change from baseline in CAT; the evaluation of the predictive value of blood eosinophil count for therapy success; and safety and tolerability (in terms of adverse events).

### Sample size and statistical methods

This cohort aimed to demonstrate that the time to worsening of COPD would be no longer in the LABA/LAMA FDC group than in the triple therapy group, i.e., that the one-sided, 97.5% confidence interval for the hazard ratio between the two arms did not exceed 1.2. Data from an earlier DACCORD cohort suggested that 35% of patients would experience worsening of COPD within 1 year; this translates into an exponential parameter λ of 0.43, assuming an exponential survival curve and taking ‘1 year’ as the unit of measurement. Assuming 20% of patients withdrew from the study (corresponding to an exponential withdrawal ratio of 0.22), 2000 patients per arm would be necessary to answer this question with high significance (4000 patients in total). However, recruitment was slower than anticipated (although the proportion of patients withdrawing was lower than predicted), and recruitment was terminated after 1185 patients were enrolled, 361 in the LABA/LAMA FDC group. Under the assumption that 14% of patients would withdraw from the study within the 1-year follow-up (translating into an exponential drop-out-rate of 0.15), based on the above statistical calculations, a power of 28.8% would be achieved.

The primary objective was analysed using a Cox regression analysis, with cohort as fixed factor. Exacerbation rates were compared between treatment groups using a negative binomial regression model including the factors age, sex, forced expiratory volume in 1 s (FEV_1_) % predicted, CAT total score, modified Medical Research Council dyspnoea scale score, and smoking status at baseline, and number of exacerbations during the 12 months prior to entry. Changes in CAT score, the proportion of patients with a clinically relevant change from baseline in CAT total score, and the number of exacerbations per patient were compared between treatment groups using either the Wilcoxon or Chi-squared test.

The effectiveness analyses were performed on the per protocol population, which included all recruited patients who completed the end-of-study visit and at least two of the three intermediate visits, and who had no relevant deviations from the observational plan. Safety analyses were performed in the safety set, which comprised all patients with valid baseline data and post-baseline data from at least one visit.

## Results

### Participants

This study was conducted between January 2018 and January 2021. Of the 1192 patients recruited, 1185 had valid baseline data and 1124 had post-baseline data from at least one visit, 340 (30.2%) of whom had been switched to dual bronchodilation (Table 1). A total of 1022 patients completed the visit at the end of one year, 967 of whom completed at least two of the three quarterly visits with no major protocol deviations (292 and 675 receiving LABA/LAMA FDC and triple therapy, respectively). The most common reason for changing from triple therapy to a LABA/LAMA FDC was ‘patient’s wish’, followed by ‘physician prefers FDC’, although in most cases ‘other’ was selected (Table 2). The most common reasons for remaining on triple therapy were to control COPD symptoms or prevent exacerbations.

**Table 1:** Baseline demographics and disease characteristics (safety set)

	LABA/LAMA FDC (N = 340)	Triple therapy (N = 784)
Sex, n (%)		
Male	172 (50.6)	477 (60.8)
Female	168 (49.4)	307 (39.2)
Age (years), mean (SD)	69.9 (9.9)	68.8 (9.2)
Age groups, n (%)		
< 65 years	100 (29.4)	258 (32.9)
65–75 years	129 (37.9)	312 (39.8)
> 75 years	111 (32.6)	214 (27.3)
Body-mass index (kg/m^2^), mean (SD)	27.8 (5.8)	27.2 (5.5)
Duration since primary diagnosis (years), mean (SD)	6.7 (4.7)	8.0 (5.6)
Duration since primary diagnosis, n (%)		
≤ 1 year	26 (7.6)	43 (5.5)
> 1 year	305 (89.7)	707 (90.2)
Missing values	9 (2.6)	34 (4.3)
Symptoms of COPD*, n (%)		
None	0 (0.0)	0 (0.0)
Exertional dyspnoea	314 (92.4)	747 (95.3)
Dyspnoea at rest	90 (26.5)	194 (24.7)
Chest tightness/chest pain	150 (44.1)	327 (41.7)
Cough	291 (85.6)	653 (83.3)
Wheezing or grunting	104 (30.6)	266 (33.9)
Prolonged expiration	123 (36.2)	251 (32.0)
Restricted exercise tolerance	265 (77.9)	653 (83.3)
Missing values	4 (1.2)	7 (0.9)
FEV_1_ (litres), mean (SD)	1.7 (0.7)	1.5 (0.6)
FEV_1_ predicted, mean (SD)	66.9 (24.7)	57.7 (22.8)
Smoking status, n (%)		
Smoker	115 (33.8)	235 (30.0)
Ex-smoker	168 (49.4)	435 (55.5)
Non-smoker	57 (16.8)	114 (14.5)
Years smoking^†^, mean (SD)	34.4 (10.1)	35.2 (11.8)
Pack-years^†^, mean (SD)	35.2 (18.8)	36.1 (22.0)

*More than one symptom possible per patient. ^†^Only smokers and ex-smokers. *LABA* long-acting β_2_-agonist, *LAMA* long-acting muscarinic antagonist, *FDC* fixed-dose combination, *SD* standard deviation; *COPD* chronic obstructive pulmonary disease, *FEV*_*1*_ forced expiratory volume in 1 s

**Table 2:** Reason for changing or remaining on therapy (safety set)

LABA/LAMA FDC (N = 340)		Triple therapy (N = 784)	
Reason to change therapy*, n (%)		Reason to remain on therapy^†^, n (%)	
Physician prefers other inhaler	14 (4.1)	Physician prefers current inhaler(s)	61 (7.8)
Physician prefers FDC	56 (16.5)	Established control of COPD symptoms	275 (35.1)
Exacerbation(s) with current COPD medication	1 (0.3)	Good tolerability	101 (12.9)
Side effect(s) with current COPD medication	5 (1.5)	Patient prefers current inhaler(s)	34 (4.3)
Patient still symptomatic with current COPD medication	25 (7.4)	Patient's wish	37 (4.7)
Patient prefers FDC	31 (9.1)	Considered necessary for exacerbation prevention	255 (32.5)
Patient prefers other inhaler	10 (2.9)	Other main reason	12 (1.5)
Patient's wish	87 (25.6)	Missing values	9 (1.1)
Other main reason	96 (28.2)		
Missing values	15 (4.4)		

*Only for patients in the LABA/LAMA FDC treatment group. ^†^Only for patients in the triple therapy treatment group. *LABA* long-acting β_2_-agonist, *LAMA* long-acting muscarinic antagonist, *FDC* fixed-dose combination, *COPD* chronic obstructive pulmonary disease

Recruitment into this cohort of DACCORD was not limited to specific molecules: 439 patients were receiving indacaterol/glycopyrronium (246 [72.4%] and 193 [24.6%] in the LABA/LAMA FDC and triple therapy groups respectively). The majority of patients continued with the same treatment regimen for the duration of follow-up (87.7% and 89.3% of patients, respectively). There was a change in regimen for 2.4% and 2.2% of patients; 9.9% of patients in the LABA/LAMA FDC group returned to their prior medication.

### Outcomes

#### Worsening of COPD

**Fig. 1:**
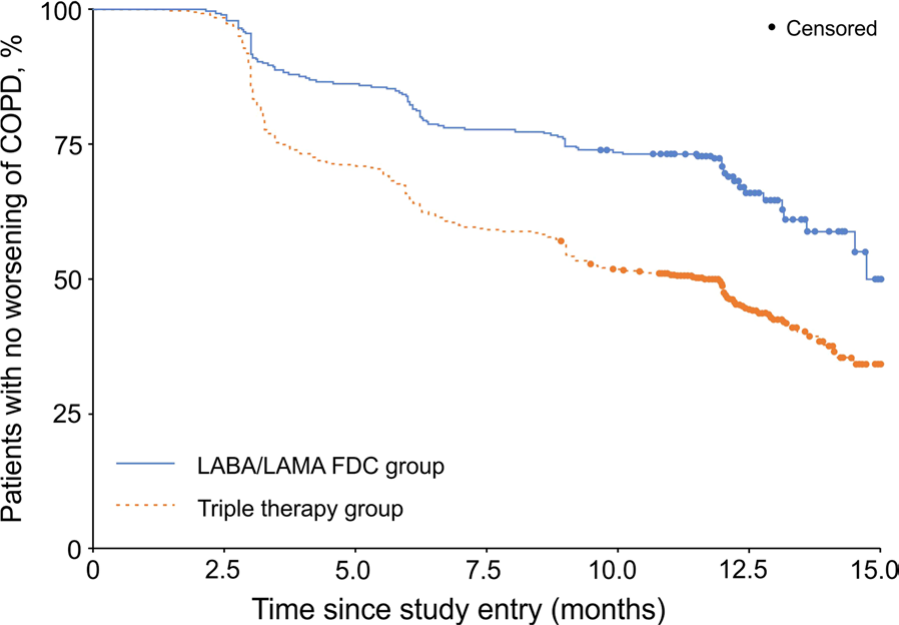
Kaplan–Meier curve for time until worsening of COPD according to exacerbations or CAT (per protocol set). *COPD* chronic obstructive pulmonary disease, *CAT* COPD Assessment Test, *LABA* long-acting β_2_-agonist, *LAMA* long-acting muscarinic antagonist, *FDC* fixed-dose combination

A lower percentage of patients in the LABA/LAMA FDC group had a worsening of COPD than in the triple therapy group (32.5% vs 55.7% at the study end; Table 3). For the primary endpoint, the time to worsening, there was a significant extension in the LABA/LAMA FDC group (hazard ratio vs triple therapy group 2.004; p < 0.001; Table 3). However, as fewer than 50% of patients had a worsening in this group it was only possible to calculate the median value in the triple therapy group (Table 3 and Fig. 1). Both of the components (i.e., CAT and exacerbations) contributed to this overall result, with a lower percentage of patients in the LABA/LAMA FDC group having a worsening based on CAT alone or exacerbations alone, and both times to worsening significantly extended (Table 3).

**Table 3:** Cumulative number (percentage) of patients experiencing a worsening of COPD by visit, and median time to worsening (per protocol set)

	LABA/LAMA FDC (N = 292)	Triple therapy (N = 675)
**Worsening of COPD according to exacerbations or CAT**		
Cumulative number (percentages) of patients experiencing a worsening of COPD by visit		
Visit 1 (after approx. 3 months)	40 (13.7)	192 (28.4)
Visit 2 (after approx. 6 months)	64 (21.9)	277 (41.0)
Visit 3 (after approx. 9 months)	79 (27.1)	330 (48.9)
Visit 4 (after approx. 12 months)	95 (32.5)	376 (55.7)
Median time until worsening of COPD [95% CI] in months	Not analysed	11.93 [9.17; 12.19]
Cox regression model, LABA/LAMA FDC vs triple therapy, hazard ratio [95% CI]; p value	2.004 [1.600; 2.512]; < 0.001	
**Worsening of COPD according to CAT**		
Cumulative number (percentages) of patients experiencing a worsening of COPD by visit		
Visit 1 (after approx. 3 months)	32 (11.0)	149 (22.1)
Visit 2 (after approx. 6 months)	46 (15.8)	213 (31.6)
Visit 3 (after approx. 9 months)	54 (18.5)	258 (38.2)
Visit 4 (after approx. 12 months)	63 (21.6)	290 (43.0)
Median time until worsening of COPD [95% CI] in months	Not analysed	14.23 [13.93; –]
Cox regression model, LABA/LAMA FDC vs triple therapy, hazard ratio [95% CI]; p value	2.223 [1.692; 2.921]; < 0.001	
**Worsening of COPD according to exacerbations**		
Cumulative number (percentages) of patients experiencing a worsening of COPD by visit		
Visit 1 (after approx. 3 months)	15 (5.1)	70 (10.4)
Visit 2 (after approx. 6 months)	27 (9.2)	113 (16.7)
Visit 3 (after approx. 9 months)	39 (13.4)	151 (22.4)
Visit 4 (after approx. 12 months)	49 (16.8)	190 (28.1)
Median time until worsening of COPD [95% CI] in months	Not analysed	Not analysed
Cox regression model, LABA/LAMA FDC vs triple therapy, hazard ratio [95% CI]; p value	1.723 [1.258; 2.360]; < 0.001	

*COPD* chronic obstructive pulmonary disease, *LABA* long-acting β_2_-agonist, *LAMA* long-acting muscarinic antagonist, *FDC* fixed-dose combination, *CAT* COPD Assessment Test, *CI* confidence interval

#### COPD exacerbations

**Fig. 2:**
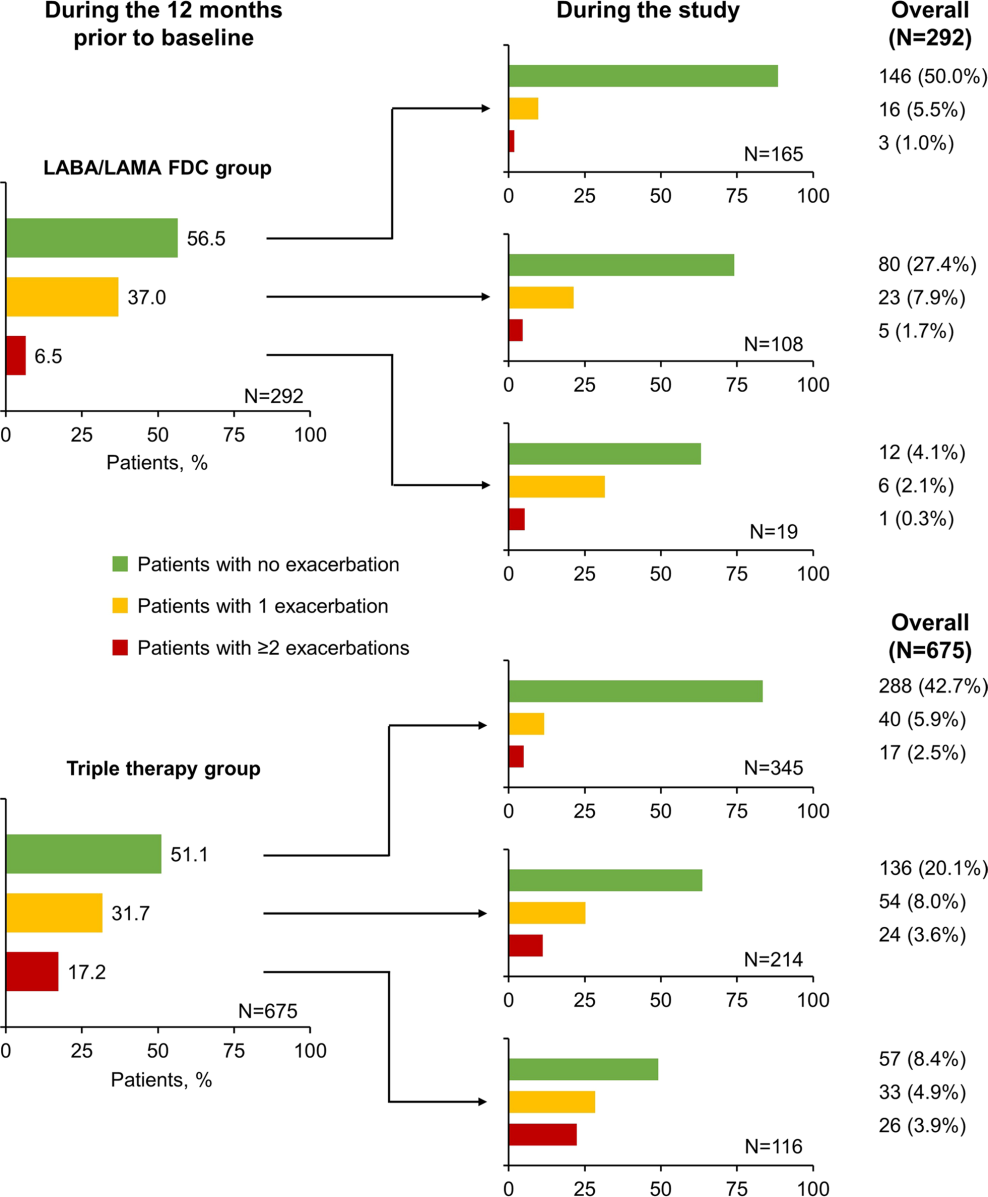
Proportion of patients by COPD exacerbation category, prior to study and during the study (per protocol set). *COPD* chronic obstructive pulmonary disease, *LABA* long-acting β_2_-agonist, *LAMA* long-acting muscarinic antagonist, *FDC* fixed-dose combination

On entry to the study, the proportion of patients who had ≥ 1 COPD exacerbation in the previous 12 months did not differ between the two groups, although 5.2% of patients in the triple group had a history of ≥ 3 exacerbations, compared with none in the LABA/LAMA FDC group (Table 4). During the study, a significantly lower proportion of patients in the LABA/LAMA FDC group exacerbated (18.5% vs 28.7%; p < 0.001), with a resultant lower annualised exacerbation rate than in the triple therapy group (0.212 vs 0.436 exacerbations/patient/year). As shown in Fig. 2, 50% of patients in the LABA/LAMA FDC group did not exacerbate during either the previous 12 months or throughout the study; 42.7% of patients in the triple therapy group met this criterion. Of those that did have a history of exacerbations, the majority did not exacerbate during the study; of note, even among patients with a history of ≥ 2 exacerbations more than half did not exacerbate during the study.

**Table 4:** COPD exacerbations, prior to and during the study (per protocol set﻿)

	LABA/LAMA FDC (N = 292)	Triple therapy (N = 675)
**During the 12 months prior to baseline**		
Patients with ≥ 1 COPD exacerbation, n (%)	127 (43.5)	330 (48.9)
p value	0.123	
Number of exacerbations, n (%)		
0	165 (56.5)	345 (51.1)
1	108 (37.0)	214 (31.7)
2	19 (6.5)	81 (12.0)
≥ 3	0 (0.0)	35 (5.2)
**During the study**		
Patients with ≥ 1 COPD exacerbation, n (%)	54 (18.5)	194 (28.7)
p value	< 0.001	
Number of exacerbations, n (%)		
0	238 (81.5)	481 (71.3)
1	45 (15.4)	127 (18.8)
2	9 (3.1)	43 (6.4)
≥ 3	0 (0.0)	24 (3.6)
Annualised exacerbation rate during the study (95% confidence limit)	0.212 (0.164, 0.274)	0.436 (0.375, 0.507)

*COPD* chronic obstructive pulmonary disease, *LABA* long-acting β_2_-agonist, *LAMA* long-acting muscarinic antagonist, *FDC* fixed-dose combination

#### COPD assessment test

On entry, the median CAT total score was slightly but significantly higher (i.e., worse) in the LABA/LAMA FDC group than the triple therapy group (Table 5). At all four visits during the study, patients in the LABA/LAMA FDC group had a greater improvement from baseline in CAT total score, both in terms of the median change from baseline and the proportion of CAT responders, with 58.2% having a clinically relevant improvement from baseline at the end of follow-up compared with 48.9% in the triple therapy group (p < 0.001).

**Table 5:** CAT total score on entry and throughout the study (per protocol set﻿)

	LABA/LAMA FDC (N = 292)	Triple therapy (N = 675)	p-value
**Median CAT score (1st, 3rd quartile)**			
Baseline visit	21.0 (13.0, 23.0)	20.0 (15.0, 25.0)	0.008
**Median CAT score, absolute change from baseline (1st, 3rd quartile)**			
V﻿isit 1 (after approx. 3 months)	− 1.0 (− 3.0 to 0.0)	− 1.0 (− 3.0 to 1.0)	0.028
Visit 2 (after approx. 6 months)	− 2.0 (− 4.0 to 0.0)	− 1.0 (− 4.0 to 1.0)	0.002
Visit 3 (after approx. 9 months)	− 2.0 (− 4.0 to 0.0)	− 1.0 (− 4.0 to 2.0)	0.004
Visit 4 (after approx. 12 months)	− 2.0 (− 5.0 to 0.0)	− 1.0 (− 4.0 to 2.0)	0.003
**CAT score responders, n (%)**			
Visit 1 (after approx. 3 months)			
Clinically relevant improvement	124 (42.5)	250 (37.0)	< 0.001
Clinically relevant worsening	32 (11.0)	149 (22.1)	
Visit 2 (after approx. 6 months)			
Clinically relevant improvement	156 (53.4)	304 (45.0)	< 0.001
Clinically relevant worsening	32 (11.0)	155 (23.0)	
Visit 3 (after approx. 9 months)			
Clinically relevant improvement	166 (56.8)	313 (46.4)	< 0.001
Clinically relevant worsening	34 (11.6)	173 (25.6)	
Visit 4 (after approx. 12 months)			
Clinically relevant improvement	170 (58.2)	330 (48.9)	< 0.001
Clinically relevant worsening	40 (13.7)	172 (25.5)	

*CAT* COPD Assessment Test, *LABA* long-acting β_2_-agonist, *LAMA* long-acting muscarinic antagonist, *FDC* fixed-dose combination, *COPD* chronic obstructive pulmonary disease

#### Predictive value of blood eosinophil count

**Fig. 3:**
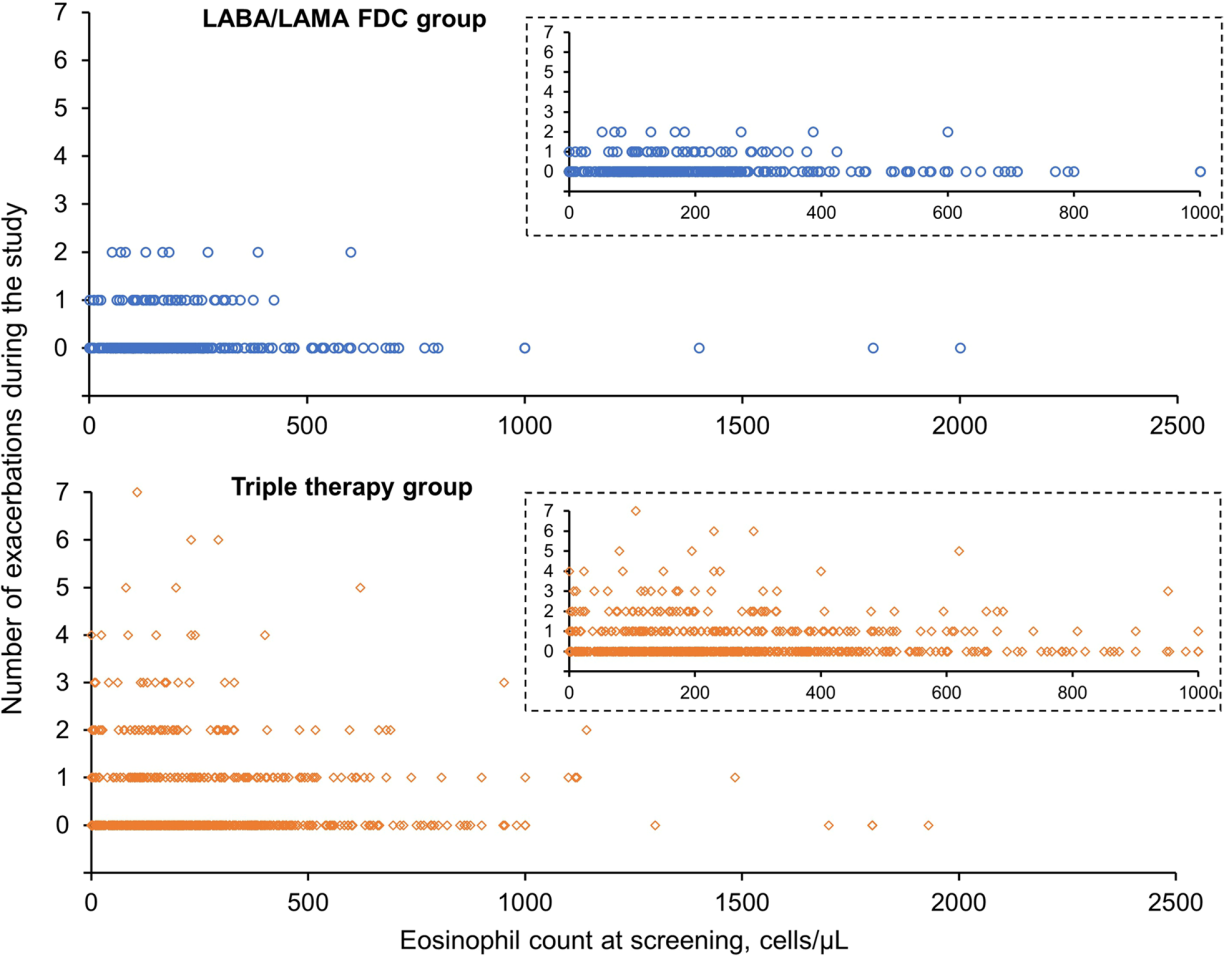
Correlation between eosinophil count at screening and the number of COPD exacerbations during the study in the LABA/LAMA FDC and triple therapy groups (per protocol set). The inset graphs display the same data as the main graphs, but with the x-axes rescaled. *LABA* long-acting β_2_-agonist, *LAMA* long-acting muscarinic antagonist, *FDC* fixed-dose combination, *COPD* chronic obstructive pulmonary disease

Although patients were eligible for entry to DACCORD if they had a blood eosinophil value assessed within 6 months prior to screening, 92.2% had values assessed within three months of screening. There was no correlation between screening eosinophil values and CAT total score, with correlation coefficients of 0.034, −0.037, and −0.093 for the relationship between eosinophil count and CAT at baseline, CAT at the end of the study, and CAT change from baseline at the end of the study, respectively. Similarly, there was no consistent relationship between eosinophil values and exacerbations, in that patients with the highest screening eosinophil values in both groups did not exacerbate during the study, whereas patients with the highest number of exacerbations had low eosinophil counts (Fig. 3). However, there was high variation in eosinophil counts in all exacerbation categories. Furthermore, in both treatment groups mean eosinophil counts were lower in patients who subsequently had a worsening of COPD than in those who did not have a worsening—although again there was high variability around the means (data not shown).

### Safety

Overall, fewer patients in the LABA/LAMA FDC group reported adverse events than in the triple therapy group (Table 6). The majority of events in both groups were non-serious and not related to study treatment. A total of 21 patients died during the study, with a lower proportion of patients in the LABA/LAMA FDC dying than in the triple therapy group (0.9% vs 2.3%); none of the fatal adverse events that occurred in the LABA/LAMA FDC group (and only five in the triple therapy group) were considered related to study treatment. The screening blood eosinophil counts for the patients who had a severe pneumonia adverse event ranged from 82 to 272 cells/µL in the LABA/LAMA FDC group (mean 174.6 cells/µL), and 3 to 620 cells/µL in the triple therapy group (mean 256.9 cells/µL).

**Table 6:** Adverse events reported during the study, overall and most common preferred term (≥ 1% in either group) (safety set)

Number (%) of patients	LABA/LAMA FDC (N = 340)	Triple therapy (N = 784)
At least one non-serious adverse event	44 (12.9)	118 (15.1)
COPD	10 (2.9)	32 (4.1)
Nasopharyngitis	4 (1.2)	16 (2.0)
Forced expiratory volume decreased	4 (1.2)	8 (1.0)
Dyspnoea	4 (1.2)	6 (0.8)
Chest discomfort	1 (0.3)	9 (1.1)
Chest pain	1 (0.3)	9 (1.1)
At least one non-serious adverse event considered related to study treatment	12 (3.5)	49 (6.3)
COPD	1 (0.3)	13 (1.7)
At least one serious adverse event	27 (7.9)	108 (13.8)
COPD	6 (1.8)	55 (7.0)
Infective exacerbation of COPD	6 (1.8)	7 (0.9)
Pneumonia	4 (1.2)	7 (0.9)
Dyspnoea at rest	2 (0.6)	15 (1.9)
Death	2 (0.6)	8 (1.0)
Cardiac failure	1 (0.3)	9 (1.1)
At least one serious adverse event considered related to study treatment	5 (1.5)	31 (4.0)
COPD	1 (0.3)	15 (1.9)

Data are number of patients (%). *LABA* long-acting β_2_-agonist, *LAMA* long-acting muscarinic antagonist, *FDC* fixed-dose combination, *COPD* chronic obstructive pulmonary disease

## Discussion

The overall aim of DACCORD Cohort 3 was to evaluate effectiveness and safety of a LABA/LAMA FDC (predominantly, although not exclusively, indacaterol/glycopyrronium) in patients with COPD who had switched from inhaled triple therapy to a LABA/LAMA FDC, in comparison to those who continued inhaled triple therapy. Importantly, the study was non-interventional (with treatment decisions not influenced by study participation), and patients received standard clinical care at primary and secondary practices throughout Germany. The majority of the baseline demographics were similar between the two groups, although patients in the LABA/LAMA FDC group were more likely to be female, aged > 75 years (the mean ages were similar) and current or non-smokers, with a trend to overall more preserved lung function.

During the follow-up period, patients switched to a LABA/LAMA FDC had overall better outcomes than those who continued inhaled triple therapy, with a lower percentage having a worsening of COPD (in terms of the overall analysis and the two components [CAT and exacerbations]), an extension in time-to-worsening, a reduction in exacerbation occurrence, and an improvement in health-related quality of life (CAT total score). Of note, the majority of patients did not exacerbate during the study, with 50.0% of those in the LABA/LAMA FDC group and 42.7% in the triple therapy group not exacerbating at all either during the year prior or during the study.

Overall, DACCORD Cohort 3 support and extend the results of WISDOM and SUNSET in confirming that ICS can be withdrawn in patients receiving inhaled triple therapy [7, 8]. These previous studies were conducted as RCTs; in contrast, the non-interventional nature of DACCORD means that the decision to de-escalate therapy was left to the investigator (presumably in discussion with the patient, given the need for informed consent on entry). The reason for therapy change or continuation was captured, and it was hoped that this would provide interesting information on standard care. In the event, the inclusion of an ‘other’ option limits the conclusions that can be drawn on the reasons for stepping down therapy, but the similarity between the two groups of the baseline demographics and disease characteristics suggest that physicians are making the decision on the basis of criteria other than purely these parameters. Of note, however, although the overall proportion of patients who had ≥ 1 COPD exacerbation in the previous year was similar in the two groups, 5.2% of patients in the triple group had a history of ≥ 3 exacerbations, compared with none in the LABA/LAMA FDC group. This suggests that patients with the most unstable disease are unlikely to be switched (which makes clinical sense).

The two previous RCTs generally demonstrated that withdrawal of ICS from inhaled triple therapy did not lead to a marked worsening in patients’ COPD. The DACCORD results not only suggest that there is no overall worsening in patients switched from triple therapy to LABA/LAMA FDC, but that LABA/LAMA FDC may be more effective than triple therapy. There are a number of potential reasons for this. First, many patients would presumably not just have the ICS withdrawn, but be switched to different LABA and LAMA molecules, since 72.4% of patients in the LABA/LAMA FDC group were receiving indacaterol/glycopyrronium FDC (at the time DACCORD was recruiting, no indacaterol-containing ICS/LABA FDC was available, and the triple therapy combination of indacaterol, glycopyrronium and the ICS mometasone was only approved in 2020—and then only for asthma). Another reason could be that adherence was improved by patients using a single LABA/LAMA FDC once-daily—most patients in the triple therapy arm were on a twice-daily regimen, with the vast majority using at least two different inhalers. This is important, as decreasing the number of daily doses and the number of different inhalers has the potential to improve medication adherence [16, 17]. However, adherence was not captured in DACCORD.

Eosinophil data were collected for all patients at screening, potentially from samples taken up to 6 months prior to study entry. This was intended to evaluate the predictive value of blood eosinophil count for subsequent outcomes. There was high variability in these counts, with a negligible correlation between screening blood eosinophil count and subsequent CAT total score, and no consistent trends in blood eosinophil count across exacerbation categories with the highest number of exacerbations during follow-up in patients with low eosinophil counts. This suggests that eosinophil values have limited utility in predicting subsequent outcomes, at least in this population that reflects the ‘real world’ patient with COPD rather than highly selected sub-populations typically recruited into RCTs. Previous RCT-based analyses suggested that ICS-containing therapy (including inhaled triple therapy) is most useful in patients with COPD who have high blood eosinophil counts (especially consistently high values [8, 18]), with a cut-point of 300 cells/µL suggested by the Global Initiative for Chronic Obstructive Lung Disease for initiation of ICS [1]. Similarly, a retrospective database analysis on the effect of ICS withdrawal versus continuation of triple therapy suggested that unsuccessful ICS withdrawal was significantly and independently associated with a blood eosinophil count ≥ 300 cells/μL [9]. Although the DACCORD analyses are not consistent with these findings, it is always challenging to compare studies of contrasting designs (especially non-interventional vs RCT vs database designs), when differences in patient characteristics can influence the results: a much higher percentage of patients entering this cohort of DACCORD had an exacerbation history than entering SUNSET (47% in DACCORD vs 34% in SUNSET) [8], and 24% of patients in the database analysis had asthma listed as a comorbidity (whereas patients with asthma were excluded from DACCORD Cohort 3) [9]. Furthermore, a limitation of the various analyses (including ours) is that they used data from a single eosinophil assessment; previous studies have shown variation in eosinophil levels in patients with COPD—both within-day variability [19] and between-day variability [20, 21]. Additional, more detailed analyses of our data are planned for the future.

The safety and tolerability data also support overall the switch from inhaled triple therapy to LABA/LAMA FDC. The proportions of patients with one or more adverse event were lower in the LABA/LAMA FDC group than the triple therapy group, with the majority of the events reported being those associated with COPD itself. Importantly, very few patients had adverse events that were considered related to study treatment, and only five patients (all in the triple therapy group) had fatal adverse events considered study treatment related.

Given this study recruited a broad, geographically diverse population being managed in primary and secondary care, the results can be generalised to patients with COPD across Germany—although only to patients receiving inhaled triple therapy. One limitation is that, as a non-interventional, observational study, the only data available are those collected from standard clinic visits, with a limited opportunity to follow-up on missing or implausible data, even if the COPD DMP in Germany does ensure regular follow-up and consistent routine care of these patients. In addition, consistent with the non-interventional status of the study, all data are collected from the study centres’ own equipment and laboratories.

In conclusion, these results suggest that physicians are able to identify patients who can be ‘stepped down’ from inhaled triple therapy to a LABA/LAMA FDC in daily routine practice (although not necessarily based on easily identifiable demographic or disease characteristics). When such patients have their therapy stepped down, there was no overall decline in their disease—and some patients had a benefit in terms of exacerbations, health-related quality of life, or overall disease control.

## Data Availability

Anonymised patient data can be requested for further research by submitting a study proposal to www.clinicalstudydatarequest.com.

## Abbreviations

CAT: COPD Assessment Test
CI: Confidence interval
COPD: Chronic obstructive pulmonary disease
DMP: Disease Management Program
FDC: Fixed-dose combination
FEV_1_: Forced expiratory volume in 1 s
ICS: Inhaled corticosteroid
LABA: Long-acting β_2_-agonist
LAMA: Long-acting muscarinic antagonist
RCT: Randomised controlled trial
SD: Standard deviation
SE: Standard error
